# Subcutaneous Immunotherapy (SCIT) with the New Polymerized Molecular Allergoid Alt a1: A Pilot Study in Children with Allergic Rhinitis Sensitized to Alternaria Alternata

**DOI:** 10.3390/jcm12134327

**Published:** 2023-06-27

**Authors:** Giulia Brindisi, Alessandra Gori, Caterina Anania, Ivana Martinelli, Martina Capponi, Giovanna De Castro, Anna Maria Zicari

**Affiliations:** 1 Department of Mother-Child, Urological Science, Sapienza University of Rome, 00161 Rome, Italy; alessandra.gori85@gmail.com (A.G.); caterina.anania@uniroma1.it (C.A.); ivana.martinelli@uniroma1.it (I.M.); m.capponi@uniroma1.it (M.C.); giovanna.decastro@uniroma1.it (G.D.C.); annamaria.zicari@uniroma1.it (A.M.Z.); 2 Department of Translational and Precision Medicine, Sapienza University of Rome, 00185 Rome, Italy

**Keywords:** Alternaria Alternata, Alt a1, Modigoid, subcutaneus immunotherapy, precision medicine, allergic rhinitis (AR), nasal FeNO (nFeNO), nasal citology, anterior active rhinomanometry (AAR), children

## Abstract

Background: We followed the effects of a new SCIT with a chemically polymerized allergen Alt a1, evaluating the trend of clinical and functional parameters in an observational-prospective study. Methods: 42 children with AR and intermittent asthma sensitized to A.A.: 17 patients started SCIT (Modigoid^®^), and 25 continued symptomatic therapy. At the initial visit (T0), all patients performed total IgE (tIgE) and specific IgE (sIgE) for Alt a1, nasal nitric oxide (nFeNo), nasal cytology, anterior active rhinomanometry (AAR) and spirometry. After 24 months (T1), they repeated the same procedures as in T0. Results: Patients treated with Modigoid presented a statistically significant (*p* < 0.001) reduction of nFeNO (T0:1651.06 ± 149.18; T1: 1394.12 ± 108.98), tIgE (T0: 311.48 ± 144.18; T1: 164.73 ± 50.69), sIgE for Alt a1 (T0: 28.59 ± 12.69; T1: 19.54 ± 7.37), an improvement of nasal airflow (T0: 71.62 ± 8.66; T1: 95.12 ± 5.91), nasal eosinophils (T0: 20.59 ± 2.35; T1: 14.88 ± 1.65) and FEV1 (T0: 95.58 ± 7.91; T1: 116.64 ± 5.94). Conclusions: The new SCIT for Alt a1 significantly improves AR symptoms from a subjective, objective point of view and laboratory and functional parameters.

## 1. Introduction

Allergy seems to be at the forefront of respiratory health, worsening with an exponential increase in allergic respiratory diseases worldwide. It is being termed an “allergic epidemic” [1]. Compared with other aeroallergens, the impact of sensitization to fungal allergens on respiratory health, among which Alternaria Alternata (A.A.) is the most important representative, has received little attention, especially in pediatric age, although characterized by molecular, pathogenic, and clinical severity peculiarities [2,3,4,5].

To date, allergy to A.A. is becoming a global health problem considering that its prevalence has reached 12.9% in the USA, where it is regarded as the “American mite”, and 8.9% in Europe [6].

Contemporary prevalence estimates are not available for Italy, and historic values likely underestimate the prevalence given the global increase in A.A. allergy prevalence. A.A. is a saprophytic mould whose spores disseminate in areas with a typical Mediterranean climate with a peak during late summer and early autumn. The saprophytic character and the ability to adapt to a wide range of climatic conditions allow this species to have a universal distribution. It can exceed 7000 conidia/m^3^ in some geographical areas, and we know that the concentration threshold capable of causing allergic symptoms has been set between 80 and 300 conidia/m^3^ [7]. Furthermore, the sensitization to A.A. appears to trigger the development of co-sensitization to other allergen sources that can exacerbate allergic symptoms [8,9,10]. Thus, exposure to this allergen can cause IgE-mediated disorders such as AR, asthma, and atopic dermatitis (AD) [11].

Asthma is one of the most severe diseases associated with A.A., considered a significant factor in both the development and exacerbations of this pathology, especially in children. Moreover, all these pathologies, even if they do not endanger the life of patients, can nevertheless cause considerable morbidity and low quality of life (QoL) in children and their families [12]. In particular, AR and asthma are atopic diseases commonly, sharing a similar etiopathogenesis, characterized by a T-helper 2 (Th2) inflammation with a release of many biomarkers [12]. This response to allergens results in IgE-mediated activation of mast cells, basophils, and eosinophils and is well-known.

The consequence is the inappropriate release of instantaneous inflammatory mediators, such as histamine, nitric oxide (NO), protease production, leukotrienes, and cytokines [13]. Suppression of this inflammatory cascade is the cornerstone of conventional pharmacotherapeutic approaches.

New pharmacological approaches, rather than aiming exclusively at improving allergic symptoms, focus on acting on the immune system to reduce the progression of the atopic march. Precision and personalized medicine are particularly suitable for these pathologies blocking a specific and pathogenic mechanism with the possibility of complete therapeutic success.

Identifying single allergenic components purified natural or recombinant, such as Alt a1, and the consequent development of molecular diagnostics of allergy were the first tools that allowed personalized diagnosis. Achieving greater diagnostic accuracy translates into significantly evolving customized therapies that have improved efficacy, working on the primary cause of allergic sensitization. Allergen-specific immunotherapy (AIT) decreases symptoms and provides long-term tolerance to the selected allergen by acting as a disease-modifying therapy. The administration, for a defined period, of increasing doses of a particular allergen to which a patient is sensitized leads to the impairment of IgE production, the increased release of IgG4, the reduction of T helper cells 2, and related cytokines (IL4, IL5, and IL13), increased T and B regulatory cells, and expression of suppressor molecules (e.g., IL-10, IL-35, TGF-β, PD-1, and CTLA-4) [14,15,16]. Thus, AIT acts on the causes of allergies and modifies its natural history.

AIT with A.A. extracts is highly effective in causing significant clinical improvement, as shown in several published studies [17,18,19,20,21]. A critical step is selecting the type of extract in AIT in terms of quality, purified allergen content, and overall allergenic activity in combination with improving the production level. Inadequate allergen dosage, adverse effects, and treatment duration are the leading causes of treatment failure [22]. The potential of AIT in treating allergic diseases is recognized as significantly superior to standard therapies.

Studies on AIT for A.A. are not uniform, considering the route of AIT administration (subcutaneous or sublingual), the type of allergen extract, and the treatment protocol [22,23]. Moreover, studies based on the most crucial molecular allergen of A.A., Alt a1, rather than the complete allergen extract, are also very few [20,21]. In many cases, Alt a 1 in its native form often elicited reactions to it; for this reason, we have evaluated the therapy with chemically modified and polymerized Alt a 1 capable of reducing the side effects of AIT and of enhancing its clinical benefits, especially in pediatric age where no studies have been conducted with this new allergen.

Therefore, we have performed a pilot study on patients with persistent AR and intermittent asthma caused by A.A. who underwent subcutaneous immunotherapy (SCIT) with the polymerized molecular allergoid of Alt a1 to evaluate clinical, functional, and laboratory parameters.

## 2. Materials and Methods

### 2.1. Study Population

This observational-prospective pilot study was focused on patients monosensitized to A.A., affected by AR and intermittent asthma, aged between 6 and 18 years old, enrolled during the ordinary clinical practice at the Pediatric Allergy Unit of the Department of Mother Child Urological Science, Sapienza University of Rome.

Patients were offered Modigoid^®^ Alt a1 in addition to symptomatic therapy. This approach consisted of the use of nasal corticosteroid (Beclometasone Dipropionate 100 mcg) 1 puff per nostril once a day, and in case of bronchospasm, bronchodilator spray (Salbutamol spray 100 mcg) and inhaled corticosteroid. SCIT was started in December 2020, outside the season in which the spores of A.A. reach their highest concentration, from May to November in Italy.

Inclusion criteria were the confirmed presence of a clinical history related to the exposure to this allergen, positive skin prick test (SPT) ≥ 3 mm only for A.A., and the presence of specific molecular IgE Alt a1 tested by Immuno-CAP.

Exclusion criteria encompassed the presence of uncontrolled asthma, severe chronic respiratory disease, and children who have undergone immunotherapy for any aeroallergen in the past or were undergoing it at the time of the case study.

SCIT with Modigoid^®^ Alt a1 was proposed during the routine clinical practice, following the EAACI recommendations for using SCIT with aeroallergens [24]. At the initial visit (T0), all the patients underwent a blood sample for the dosage of total IgE (tIgE), specific IgE for A.A. (Alt a1); moreover, they performed nasal nitric oxide (nFeNo), nasal cytology, basal and post decongestion anterior active rhinomanometry (AAR) and a basal and post bronchodilatation spirometry. The subjective sensation of nasal obstruction was evaluated through Nasal Symptom Score (NSS) [25]. All bronchial functionality and relative parameters have been examined and will be the main topic of further studies and scientific insights. Every six months, all patients were evaluated in an outpatient setting with regular follow-up visits.

After 24 months of SCIT (T1), all patients underwent the same procedures as at the enrollment visit (T0) and were also asked if they had reduced the use of standard therapy during the last 24 months. Parents or guardians of all enrolled patients signed a written informed consent. No clinical diagnostic interventions performed on patients differed from the standard clinical practice. The diagnosis of AR and its characteristics in terms of frequency (intermittent or persistent) and intensity (mild or moderate/severe) was made according to ARIA guidelines [26]. According to GINA guidelines, the same was made for asthma [27]. This pilot study was performed according to the Declaration of Helsinki regarding biomedical research involving human subjects, and the study protocol was approved by the local ethics committee of the Sapienza University of Rome (number of protocol: 0441/2023).

### 2.2. Serological Biomarkers

Serum total IgE levels and specific molecular IgE levels to A.A. (Alt a1) were tested using a fluorescence enzyme immunoassay (FEIA) with capsulated cellulose polymer solid phase (Immuno CAP^®^) coupled allergens (Thermo Fisher Scientific Inc, Phadia AB, Uppsala, Sweden). Results were expressed in kU/L, and a cut-off point of 0.35 kU/L stands for positivity [28].

### 2.3. Nasal Nitric Oxide (nFeNO)

The nasal FeNO (nFeNO) technique using a tight facemask with a fixed flow connected to an analyzer (Medisoft nFeNO analyzer, Medisoft s.r.l., Naples, Italy) allowed the measurement of nFeNO according to the American Thoracic Society/European Respiratory Society guidelines (ERS/ATS). During inspiration up to total lung capacity, patients inhale through the nose from the NO-free analyzer and exhale through disposable nose pads at a constant flow of 350 mL/s for 60 s. [29,30].

### 2.4. Mean Nasal Flow (mNF) at AAR

AAR is an easy technique that takes about 10 min, allowing the detection of mean nasal flow (mNF) and nasal resistance to the air passage. It was performed according to the ICSR (Committee for the Standardization of Rhinomanometry) guidelines using a RINOPOCKET ED200 (EUROCLINIC^®^, Imola, Bologna, Italy) [31]. Rhinomanometer enables us to measure the nasal flow (cm^3^/s) in the right and left nostrils at a pressure of 150 Pascal in both inspiration and expiration. The obtained values were compared with the pediatric ones’ height dependent. Nasal obstruction was classified, using a basal AAR, as reported below: very severe if airflow values were less than 29% of the predicted ones, severe if the values were between 29% and 42%, moderate if between 43% and 56%, mild between 57% and 70% and absent for values above 71% [32]. Additional information was provided using a sympathetic alpha mimetic such as Naphazoline Nitrate nasal spray (Rinazine^®^ 100 mg/100 mL), one puff in each nostril. After 15 min, an AAR post-decongestion was repeated to evaluate the modification of the nasal flows [33].

### 2.5. Nasal Eosinophils Count at Nasal Cytology

Nasal cytology is a simple and safe diagnostic procedure that is easily performed by a small plastic curette (nasal scraping) at the middle portion of the inferior turbinate. Once the sampling has been conducted, the cellular material is spread on a glass slide, then fixed by air drying and subsequently stained according to the May Grunwald-Giemsa (MGG) method. This staining method allows highlighting all the cellular components of the nasal mucosa, the cells of immune-phlogosis (neutrophils, eosinophils, lymphocytes, and mast cells), bacteria, mycotic spores, and fungal hyphae. The observation of the slide is carried out using a standard optical microscope at 1000×. We read by fields (minimum 50) to analyse the rhino-cytogram to find more cellular elements. In particular, we were interested in the nasal eosinophils count [34].

### 2.6. Nasal Symptom Score (NSS)

NSS is a written, four-item questionnaire that assesses the most frequently reported rhinitis symptoms, such as nasal obstruction, rhinorrhea, itching, and sneezing, referring to the two weeks before the study. The answer to each item presents a score ranging from 0 to 3 (0, absent; 1, weak; 2, moderate; 3, severe). The sum of the total indicates the severity of rhinitis with a maximum score of 12 [25]

### 2.7. Forced Expiratory Volume in the First Second (FEV1) at Spirometry

FEV1 is the most critical parameter used to detect bronchial obstruction through spirometry. It was measured with a Cosmed Spirometer (Cosmed, Rome, Italy), according to ATS/ERS procedures. Basal FEV1 was expressed as a percentage of predicted average values adjusted for height, gender, and ethnicity. In addition to basal spirometry for each patient, we performed a spirometry post-bronchodilatation with Salbutamol spray 100 mcg, according to ATS/ERS procedures [29,35].

### 2.8. Schedule of Modigoid Administration

Regarding the method of administration, SCIT with purified polymerized Alt a 1 (Modigoid^®^ Alt a 1, ROXALL Medicina España S.A., Zamudio, Spain) required a rush build-up of 0.2 mL (0.8 μg of Alt a 1) + 0.3 mL (1.2 μg of Alt a 1) separated by 15-min. Then, patients received a maintenance dose of 0.5 mL (2 μg of Alt a 1) administered once a month till the end of the treatment. The entire treatment duration is 3–5 years. Still, the patients were re-evaluated after the first 24 months of therapy with an outpatient checkup and by repeating the same procedures as in T0.

### 2.9. Statistical Analysis

Statistical analysis was performed using IBM SPSS version 23.0 (SPSS, Chicago, IL, USA). For the continuous variables, the Shapiro-Wilk normality test was performed; these continuous quantities were represented by mean value and standard deviation (SD). Nominal and ordinal variables were described in terms of relative counts and frequencies. The comparison of the assumed clinical values–both for the treatment and control group–at baseline (T0) and follow-up (T1) was performed by “paired-samples T-test” and by “Wilcoxon signed-rank test for paired samples”. The comparison between the treatment and control group, both at T0 and at T1, was performed by “T-test for unpaired samples” and “U Mann Withney test”. The existence of a relationship between immunotherapy and the less use of symptomatic drugs has been investigated using the “Chi-Square test”. In all cases, a *p*-value ≤ 0.05 was considered statistically significant.

## 3. Results

Forty-two patients, all allergic to A.A., agreed to be enrolled in this study. Among them, 25 continued only standard therapy (control group), and 17 started Modigoid^®^ Alt a1 (treatment group) in addition to symptomatic drugs. All the enrolled patients presented moderately persistent AR and mild intermittent asthma. The characteristics of the study population are reported in Table 1 below.

**Figure 1:**
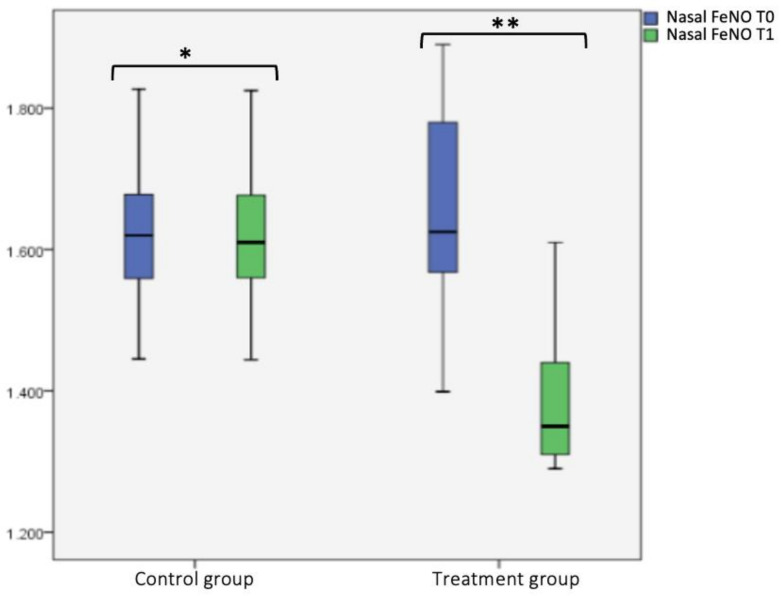
Nasal FeNO (nFeNO) at T0 and T1 in the treatment and control group. Box plot representing nFeNO values at T0 and T1 in both the treatment and control group (* not significant, ** significant).

Considering the intragroup variability, we did not find any statistical significance for the control group’s nFeNO at T0 and T1. Instead, for the treatment group, we found a statistical significance difference between nFeN0 at T0 (1651.06 ± 149.18) and T1 (1394.12 ± 108.98) (*p* < 0.001), as shown in box plot Figure 1 and Table 2. Considering the intergroup variability, we did not find a statistically significant difference between the nFeNO of the treatment or control group at T0; this difference was found at T1 (*p* < 0.001), as shown in the box plot Figure 1 and Table 2.

**Figure 2:**
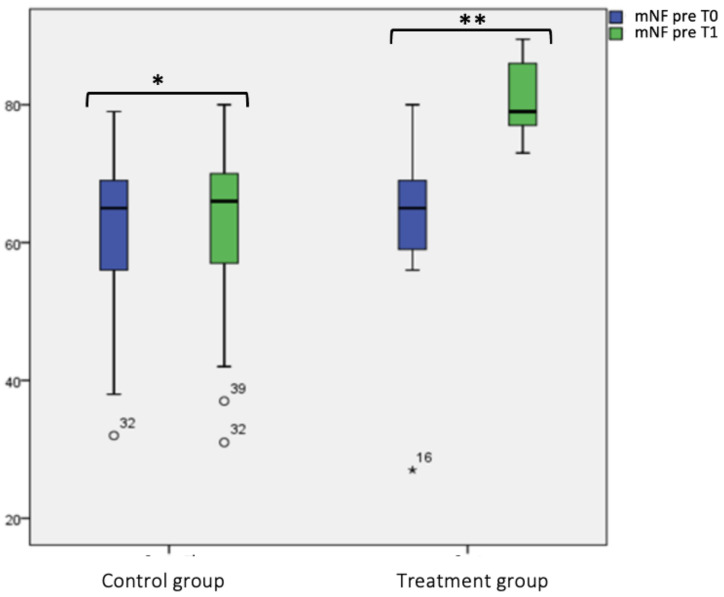
Mean nasal flow (mNF) pre-hydrazine at T0 and T1 in the treatment and control group. Box plot representing mean nasal flow (mN)F pre-hydrazine at T0 and T1 in both treatment and control group (* not significant, ** significant).

**Figure 3:**
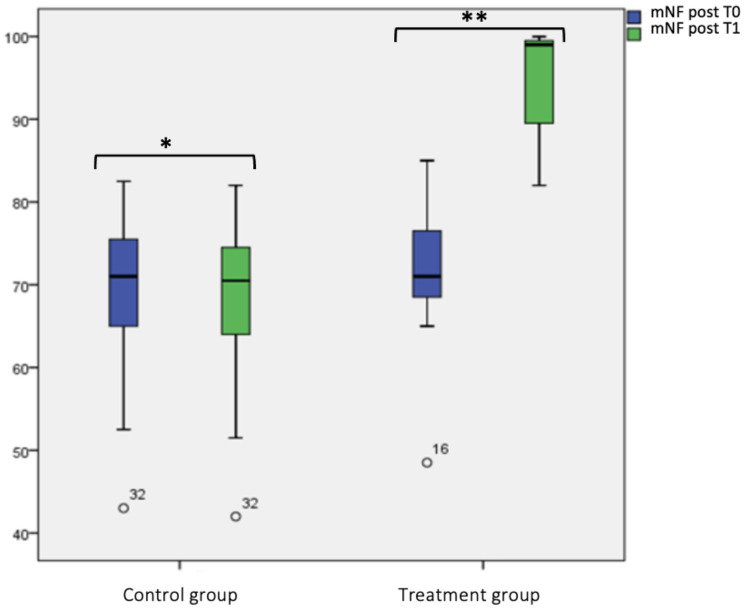
Mean nasal flow (mNF) post-hydrazine at T0 and T1 in the treatment and control group. Box plot representing mean nasal flow (mNF) post-hydrazine at T0 and T1 in both treatment and control group (* not significant, ** significant).

Considering “mNF pre-hydrazine” and “mNF post-hydrazine”, we did not find any statistical significance at T0 and T1 for the control group. Instead, for the treatment group, we found a statistical significance difference between “mNF pre-hydrazine” at T0 (63.68 ± 12.23) and T1 (80.88 ± 5.54) (*p* < 0.001), as shown in box plot Figure 2 and Figure 3 and Table 2. In the same group, also between “mNF post-hydrazine” at T0 (71.62 ± 8.66) and T1 (95.12 ± 5.91), it was found a statistical significance (*p* < 0.001), as shown in box plot Figure 2. Considering the intergroup variability, we did not find a statistically significant difference for both “mNF pre- and post-hydrazine” between the treatment and control group at T0; this difference was found at T1 (*p* < 0.001), as shown in box plot Figure 2 and Figure 3 and Table 2.

**Figure 4:**
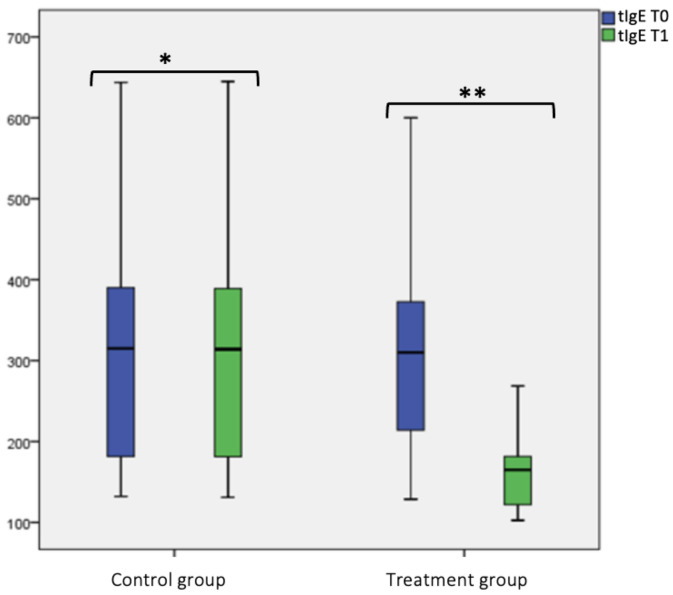
Total IgE (tIgE) level at T0 and T1 in the treatment and control group. Box plot representing total IgE (tIgE) level at T0 and T1 in both treatment and control group (* not significant, ** significant).

The same for tIgE: no statistical difference in the control group at T0 and T1, instead in the treatment group, we found a statistical significance difference (*p* < 0.001) between tIgE at T0 (311.48 ± 144.18) and T1 (164.73 ± 50.69), as shown in box plot Figure 4 and Table 2. Considering the intergroup variability, we did not find a statistically significant difference in tIgE level between the treatment and control group at T0; this difference was found at T1 (*p* < 0.001), as shown in Figure 4 and Table 2.

**Figure 5:**
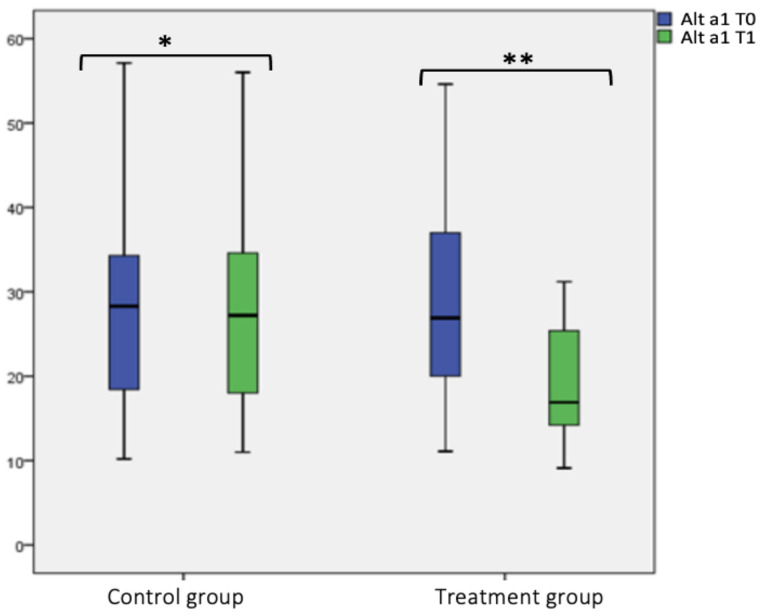
Alt a1 level at T0 and T1 in the treatment and control group. Box plot representing Alt a1 level at T0 and T1 in both treatment and control group (* not significant, ** significant).

Considering the specific IgE for Alt a1, no statistical difference was found in the control group at T0 and T1. In the treatment group, we found a statistical significance difference between Alt a1 at T0 (28.59 ± 12.69) and T1 (19.54 ± 7.37) (*p* < 0.001), as shown in the box plot Figure 5 and Table 2. Considering the intergroup variability, we did not find a statistically significant difference for Alt a1 level between the treatment and control group at T0; this difference was found at T1 (*p* < 0.001), as shown in Figure 5 and Table 2.

**Figure 6:**
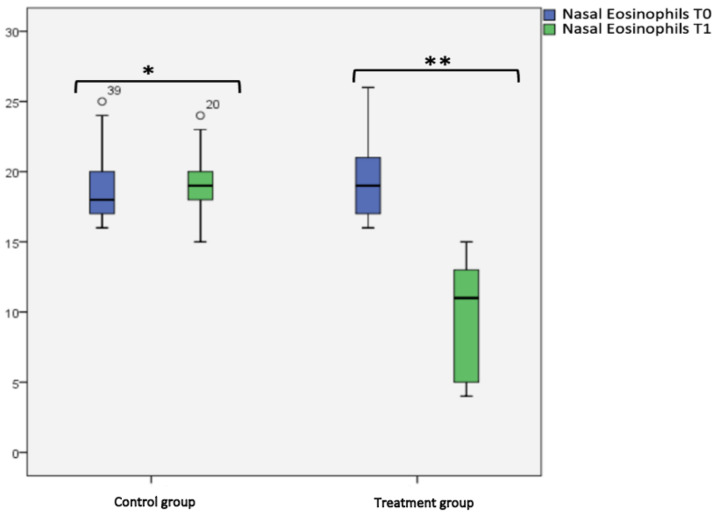
Nasal eosinophils at T0 and T1 in the treatment and control group. Box plot representing nasal eosinophils at cytology at T0 and T1 in both treatment and control group (* not significant, ** significant).

For nasal cytology, we considered the percentage of eosinophil cells. No statistical difference was found in the control group at T0 and T1. In the treatment group, we found a statistical significance at T0 (19.47 ± 2.87) and T1 (9.65 ± 4.20) (*p* < 0.001), as shown in box plot Figure 6 and Table 2. Considering the intergroup variability, we did not find a statistically significant difference in the percentage of eosinophil cells between the treatment and control group at T0; this difference was found at T1 (*p* < 0.001), as shown in Figure 6 and Table 2.

In the end, considering nasal symptom score, no statistical difference was found in the control group at T0 and T1. In the treatment group, we found a statistical significance (*p* < 0.001) at T0 (20.59 ± 2.35) and T1 (14.88 ± 1.65). Considering the intergroup variability, we did not find a statistically significant difference in NSS between the treatment and control group at T0; this difference was found at T1 (*p* < 0.001), as shown in Table 2.

Moreover, all the patients performed both basal spirometry (FEV1 pre) and post-bronchodilation spirometry (FEV1 post). It was found a significant difference between “FEV1 pre-bronchodilation” before and after 24 months of therapy in the treatment group not in the control one, as well as between “FEV1 post” at T0 and T1. The mean “FEV1 pre” at T0 was 85.7 ± 5.15, instead the mean “FEV1 pre” at T1 was 100.23 ± 1.34 (*p* < 0.001). The mean “FEV1 post” at T0 was 95.58 ± 7.91, instead the mean “FEV1 post” at T1 was 116.64 ± 5.94. After applying the Wilcoxon test, we observed a statistically significant difference between the mean “FEV1 pre” at T0 and T1 (*p*< 0.001), and between the mean “FEV1 post” at T0 and T1 (*p* < 0.001). Furthermore, a statistically significant difference was found for both FEV1 pre and post-between treatment and control group at T1 (*p* < 0.001). All these data are reported in Table 2.

In addition, we evaluated after 24 months, the need for standard therapy both for the treatment and control groups. After applying a Chi-Square test, we observed a statistically significant reduction in the use of standard therapy in the treatment group in comparison with the controls at T1 (*p* < 0.001). We underline that during the entire study duration, we did not report any adverse reactions in our study population reinforcing the concept of the safety of this new SCIT.

## 4. Discussion

The allergenicity and clinical effect for Modigold were demonstrated to be encouraging, providing significant improvements in AR symptoms both from a subjective and objective point of view as well as considering laboratory and functional parameters. After only 24 months of treatment with this newly polymerized allergoid, the beneficial clinical effect of SCIT was reported as effective, with no adverse events associated. Moreover, the reduced use of standard therapy should be underlined among patients treated with the new SCIT.

Specific allergen immunotherapy is currently the only treatment capable of modifying the entire immune system by reducing the reactivity towards single allergens due to a down-regulation of the Th2 response [24]. Moreover, SCIT exerts a fundamental role as a preventive toll of severe respiratory pathologies such as asthma in patients with AR alone [28]. Another critical role of SCIT is the blocking potential of the molecular spreading towards other aeroallergens in addition to AA, and this could be much more important, especially in a pediatric population still mono-sensitized to a single aero-allergen [36]. Furthermore, in this field, precision medicine plays a pivotal role in the context of SCIT, allowing the development of individualized therapies on the allergenic profile of each patient to make the therapeutic intervention the most effective [37].

To the best of our knowledge, there are no published studies that, in pediatric age, have evaluated the effects of Modigoid Alt a1 from a subjective clinical, objective functional, and laboratory point of view. The detection of specific biomarkers, which may be response predictor indices to SCIT, is currently a proactive area of research in precision medicine.

Considering A.A., most current studies are related to AIT preparation with nAlt a1 or rAlt a1 commercial extract.

Tabar et al. have conducted a double-blind, randomized, placebo-controlled trial of subcutaneous immunotherapy with the purified major allergen Alt a 1 to evaluate the efficacy and safety of two different doses of Alt a 1 (0.2 or 0.37 mg of Alt a 1 per dose) in patients (12–65 years of age) with AR sensitized to A.A. [17]. The authors found a statistically significant reduction in symptoms and medication score with the 0.37-mg dose of Alt a1 compared to the placebo group after 12 months of therapy. Both active groups presented reduced cutaneous reactivity and IgE levels, as well as increased IgG4 levels, compared to the placebo group. No severe adverse drug events were reported, showing a similar safety profile. Therefore, immunotherapy with Alta1 was efficacious and safe, reducing symptoms and medication consumption among patients affected by AR after only one year of treatment [17].

Asturias et al. published a study where they compared the natural Alt a1 (nAlt a 1) with the recombinant one (rAlt a1) in 42 adults allergic to A.A. Results showed no IgE-binding differences between nAlt a 1 and rAlt a 1. They both elicited a similar response in SPT compared with the A.A. extract. Moreover, they demonstrated that specific IgE levels to nAlta1 or rAlta1 presented a significant correlation with similar sensitivity and specificity. So, nAlt a1 and rAlta1 are helpful for a reliable diagnosis of A.A. sensitization comparable with its extract [38].

Prieto et al. published a randomized, double-blind trial in which subjects (9–60 years of age) with AR and/or asthma sensitized to A.A. were randomized to receive a placebo (*n* = 18) or purified natural Alt a1 (*n* = 22) subcutaneous ITS for one year [20]. At baseline and after 6 and 12 months of ITS, bronchial responsiveness to adenosine 5′-monophosphate (AMP), methacholine, exhaled nitric oxide (eFeNO), exhaled breath condensate pH, and serum Alt a1-specific IgG4 antibodies were measured [20]. The authors concluded that ITS with purified nAlt a1 is well tolerated inducing an allergen-specific IgG4 response. However, this therapy is not associated with significant changes in AMP or methacholine responsiveness or improvements in inflammation markers in the exhaled air. Thus, they found a discrepancy between the ITS-induced increase of the IgG4 antibodies levels and their action on the responsiveness of bronchial airways [20].

Indeed, the recent study of Rodriguez et al., conducted among 65 patients who started immunotherapy with Alt a 1 (extract of A.A. from strain 103.33 derived by double water-soluble extraction and freeze-drying) showed that ITS with Alt a 1 is able to reduce allergic symptoms, desensitizing patients and the relative drug consumption. As a confirmation, they found, after immunoblotting the patient’s sample, the elimination of Alt a 1 sensitization in several cases [7].

To the best of our knowledge, only two studies were conducted in adults to evaluate treatment with Modigoid Alt a1.

One was made by A. Ferrer, who concluded that this treatment induced significant improvements in AR and asthma symptoms in terms of medication required by patients and safety [39]. However, this study did not investigate clinical improvement using objective functional evaluations. The assessment of treatment efficacy derives from the analysis of the specific IgE for A.A. levels and the subjective feedback of patients.

The other one was made by Morales et al., who characterized from a biochemical and immunochemical point of view the new polymerized A.A. allergoid in comparison with its extract [40]. They concluded that this new allergoid is safer than the extract due to its lower IgE binding capacity inducing tolerance through T cell shift to Treg (IL-10) while maintaining the same efficacy as no adverse events were registered. Thus, a new allergoid could be an effective and safe therapy leading to cytokine stimulation with IgG antibodies’ synthesis able to prevent IgE binding to the allergen [40].

However, all the studies cited so far, many of which have not been carried out in the pediatric age, have evaluated only the subjective improvement in clinical and laboratory parameters and only, in some cases, the reduced consumption of drugs.

So, this is the first study conducted in a pediatric population with the new allergoid Alta1, analyzing both subjective (NSS) and objective parameters (AAR, spirometry, and nasal cytology) as well as functional (naal nitric oxide) and laboratory ones (total and specific IgE for Alt a1), in children mono-sensitized to A.A.

Results from our study, have shown that after 24 months of SCIT with Modigoid, patients presented a statistically significant reduction of nFeNO levels, nasal eosinophils at cytology, tIgE, and specific IgE for Alt a1 as well as an improvement of NSS and nasal airflow. Moreover, after 24 months of therapy, it was also observed that the use of standard drugs was reduced in the group treated with Modigoid compared with controls. This general trend was also observed during the visits we organized every six months in the outpatient setting at our hospital. Another interesting trend observed in our randomly enrolled population was the prevalence of the male gender. This observation also aligns with another study on patients allergic to A.A., which found a high prevalence of young male patients affected by T2-high asthma sensitized to A.A. [41]. Therefore, we hope that further studies can be carried out in the future considering the genetic or hormonal component linked to the development of this specific allergy, given that gender seems to play a fundamental role in A.A. allergy.

Thus, a better understanding of the mechanisms underlying the pathogenetic potential of A.A. is essential to develop timed and increasingly advanced therapies from a molecular point of view. We must consider that Alt a 1 is the genuine allergen, main elicitor of the allergen process, and marker of primary sensitization identified in over 90% of patients. Despite more than two decades of studies on its molecular structure, researchers partially know its intrinsic sequence and its biological function remains unknown. The high-resolution X-ray crystal structure of recombinant Alt a 1 was recently presented. The structure reveals that Alt a 1 forms a unique, dimeric, b-barrell structure unlike any other structure currently reported in the Protein Data Bank (PDB). Therefore, Alt a 1 has a structure that has no equivalent in nature, also characterized by intramolecular disulfide bridges, which increase its stability (having IgE epitopes that can withstand the heat treatment of 958 °C) and contribute to the formation of Alt a 1 dimers. At the same time, the N-terminal cysteine covalently binds to the equivalent residue in each monomer [42]. This bridge holds two dimers in a peculiar “butterfly” configuration. Previous studies have also demonstrated the ability of Alt a 1 to form a tetrameric structure, which can be linked to the dietary flavonoid quercetin. The stability of the tetrameric Alt a1-quercetin is pH-dependent: at low ambient acidity, such as in the bronchial epithelium with a pH value of 6, it is stable, while this complex is broken down at a pH below 5.5 [42].

These and other peculiar structural characteristics of Alt a 1 certainly have several implications from the point of view of allergenicity. Some evidence indicates that the oligomerization of Alt a 1 is an essential prerequisite for its allergenic potential. Indeed, it has been observed that cross-linking of at least two FcεRI-linked IgE by an allergen is required for mast cell and basophil degranulation [43]. So, allergen concentration is a key factor in the dimerization of monomeric allergens. Previous studies have shown that many allergens can produce dimeric or oligomeric structures at higher concentrations: allergens tend to have a monomeric form at low concentrations. In comparison, they tend to dimerize or oligomerize at higher concentrations resulting in a more powerful cascade of allergic inflammation [43].

Therefore, the major limitation of native A.A. AIT is its inbuilt allergenic activity resulting primarily from its conformational epitopes detected by specific antibodies. To decrease the unpleasant collateral effects of AIT but to consider all these observations, the scientific research led to the production of allergen derivates that are generically known as allergoids also for Alt a 1. An allergoid is an edited allergen engineered to have reduced allergenicity compared to the native allergen, along with adequate immunogenicity [44]. Using allergoids in an AIT tolerance-induction strategy is rationalized by decreasing the reaginic antibodies (less allergenicity) and maintaining the IgG epitopes and linear T cell epitopes (immunogenicity). The safety and efficacy of allergoid-immunotherapy enhance it as a fundamental tool for desensitization. The lack of allergenicity of allergoids allows their use in accelerated up-dosing schemes, as well as ultra-short-course boosters, shortening the immunotherapeutic process [44]. Several desirable new properties result from the increased molecular weight of polymerized allergens. For example, a higher molecular weight cross-linked polymer of 100 allergen molecules has less surface area than the surface area of 100 individual allergen molecules. Thus, the polymer would have fewer exposed antigenic determinants to react with IgE antibodies on mast cells. A polymer of 100 allergen molecules would cross-link far fewer pairs of IgE molecules on the surface of a mast cell, and therefore polymer has less opportunity to interact with IgE-sensitized mast cells [45].

As a consequence of the above, the allergenicity of polymerized allergens would be expected to be less than that of their monomeric counterparts. By titrations of weight-equivalent amounts of monomeric and polymeric allergens, it has been confirmed that the allergenicity of polymers is approximately 1/100,000 of the weight-equivalent monomer. This consideration allows higher safe starting doses, fewer systemic reactions, and fewer injections for a course of polymer immunotherapy than for conventional immunotherapy [45]. Those are relevant advantages, especially in the pediatric population leading to a more significant number of patients desiring immunotherapy due to the reduced number of injections and the earlier onset of symptom improvement.

Indeed, an important goal to reach is compliance in patients who start AIT due to the long duration of treatment as well as the possibility of local and systemic reactions, as appears mostly in AIT with native A.A. allergen [22]. The general approach of AIT requires from 3 to 5 years of treatment. However, after 24 months, some clinical and functional improvement is already possible. The possibility with Modigoid of administering higher doses of polymerized allergens with no safety concerns helps to reach the maintenance dose in a single day with a shorter induction phase than the standard AIT with Alt a1 [21,38] weight-equivalent. Indeed, AIT with the molecular allergoid Alt a 1 is a new and powerfully effective approach for treating allergic diseases with sensitization to A.A., mainly when administered in AR to prevent the progression of the atopic march and the development of asthma. Furthermore, it is essential to spread the urgency to intervene early with AIT among pediatricians to prevent the progression of allergic diseases. Further studies are warranted to provide the potential beneficial effects of this new therapy.

### 4.1. Strength of the Study

The strengths of this work are several. First, it was a unique study in pediatric age using the new SCIT for Alt a1. Secondly, it is the only study in pediatric age that analyzes from a laboratory point of view (specific IgE dosage for Alta1), functional (nasal and bronchial nitric oxide dosage), subjective (through NSS) and objective (rhinomanometry, spirometry, and nasal cytology) children mono-sensitized to A.A. Third point: this work, by under of the encouraging results obtained, underlines the fundamental role of SCIT with this new polymerized allergen in the prevention of the development of AR comorbidities as well as of the molecular spreading toward other aeroallergens in addition to AA, with a high safety profile.

### 4.2. Limitations

Our results should be interpreted considering some limitations. First of all, the small sample size; this is a pilot study related only to a single center in Rome with size few pediatric patients enrolled. However, we should consider that this small number is due to the difficulties of finding children mono-sensitized to A.A., which are inclined to affect ITS. Therefore, we hope that a multicenter clinical trial, extended to a national and international level, will be established shortly to increase the sample size and corroborate our long-term effectiveness and safety findings.

## 5. Conclusions

This study highlighted that AIT with the new purified and polymerized allergoid of Alt a 1 used in pediatric patients appears to induce notable benefits. This type of treatment significantly improves allergic symptoms from a subjective and objective clinical point of view and laboratory and functional parameters. Therefore, an early and timely start of AIT should be increasingly offered to children to prevent complications not only among respiratory pathologies but also with desirable rises in their quality of life.

## Figures and Tables

**Table 1:** Characteristics of the study population.

Characteristic	Control Group	Treatment Group	*p*-Value
N. patients, *n* (%)	25	17	
Male	21 (84%)	15 (88.2%)	
Age, mean ± SD, years	12.25 ± 6.26	12.55 ± 2.88	0.265
Weight, mean ± SD, Kg	43.19 ± 9.22	44.55 ± 8.23	0.672
Height, mean ± SD, cm	152.36 ± 10.80	154.68 ± 10.96	0.564
Allergic Rhinitis			
Moderately persistent, *n* (%)	25 (100%)	17 (100%)	
Asthma			
mild intermittent, *n* (%)	25 (100%)	17 (100%)	

**Table 2:** Comparison of intra-group and inter-group values related to the considered variables at T0 and T1.

Characteristic	Value				*p*-Value Intragroup
	T0		T1		
	Mean ± SD	*p*-Value Intergroup	Mean ± SD	*p*-Value Intergroup	
Nasal FeNO (nFeNO)					
-Control group	1613.40 ± 104.63	*p* = 0.341	1612.84 ± 104.47	*p* < 0.001	*p* = 0.248
-Treatment group	1651.06 ± 149.18		1394.12 ± 108.98		*p* < 0.001
IgE total, Ku/L					
-Control group	311.84 ± 156.22	*p* = 0.994	311.89 ± 156.41	*p* < 0.001	*p* = 0.813
-Treatment group	311.48 ± 144.18		164.74 ± 50.69		*p* < 0.001
Alt a1, Ku/L					
-Control group	28.12± 11.46	*p* = 0.902	27.95 ± 11.38	*p* = 0.006	*p* = 0.457
-Treatment group	28.58 ± 12.69		19.54 ± 7.37		*p* < 0.001
mNF (%) pre-hydrazine					
-Control group	63.67 ± 12.23	*p* = 0.652	80.8 8± 5.54	*p* < 0.001	*p* = 0.75
-Treatment group	61.98 ± 11.65		62.04 ± 12.09		*p* < 0.001
mNF (%) post-hydrazine					
-Control group	71.61 ± 8.65	*p* = 0.8	95.12 ± 5.9	*p* < 0.001	*p* = 0.988
-Treatment group	69.12 ± 9.13		69.12 ± 9.44		*p* < 0.001
Nasal eosinophils					
-Control group	18.96 ± 2.45	*p* = 0.541	18.96 ± 2.34	*p* < 0.001	*p* = 0.995
-Treatment group	19.47 ± 2.87		9.65 ± 4.20		*p* < 0.001
Nasal symptom score					
-Control group	20.58 ± 2.34	*p* = 0.4	14.88 ± 1.65	*p* < 0.001	*p* = 0.841
-Treatment group	21.16 ± 1.77		21.2 ± 1.58		*p* < 0.001
FEV1 pre-broncodilatation					
-Control group	84.92± 3.94	*p* = 0.579	84.96 ± 4.35	*p* < 0.001	*p* = 0.846
-Treatment group	85.7± 5.16		100.23 ± 1.35		*p* < 0.001
FEV1 post-broncodilatation					
-Control group	93.84± 5.01	*p* = 0.385	93.88 ± 5.44	*p* < 0.001	*p* = 0.845
-Treatment group	95.59± 7.91		116.65 ± 5.95		*p* < 0.001

## Data Availability

Not applicable.
